# Dihalogenated nitrophenols in drinking water: Prevalence, resistance to household treatment, and cardiotoxic impact on zebrafish embryo

**DOI:** 10.1016/j.eehl.2024.02.004

**Published:** 2024-03-04

**Authors:** Hongjie Sun, Yingying Liu, Chunxiu Wu, Lena Q. Ma, Dongxing Guan, Huachang Hong, Haiying Yu, Hongjun Lin, Xianfeng Huang, Peng Gao

**Affiliations:** a Key Laboratory of Watershed Earth Surface Processes and Ecological Security, College of Geography and Environmental Science, Zhejiang Normal University, Jinhua 321004, China; b College of Chemistry and Life Science, Zhejiang Normal University, Jinhua 321004, China; c Institute of Soil and Water Resources and Environmental Science, College of Environmental and Resource Sciences, Zhejiang University, Hangzhou 310058, China; d National and Local Joint Engineering Research Center for Ecological Treatment Technology of Urban Water Pollution, College of Life and Environmental Science, Wenzhou University, Wenzhou 325035, China; e Department of Environmental and Occupational Health, and Department of Civil and Environmental Engineering, University of Pittsburgh, Pittsburgh, PA 15261, United States; f UPMC Hillman Cancer Center, Pittsburgh, PA 15232, United States

**Keywords:** Dihalogenated nitrophenols, Household water treatment, Zebrafish embryo, Reactive oxygen species, Cardiotoxicity

## Abstract

Dihalogenated nitrophenols (2,6-DHNPs), an emerging group of aromatic disinfection byproducts (DBPs) detected in drinking water, have limited available information regarding their persistence and toxicological risks. The present study found that 2,6-DHNPs are resistant to major drinking water treatment processes (sedimentation and filtration) and households methods (boiling, filtration, microwave irradiation, and ultrasonic cleaning). To further assess their health risks, we conducted a series of toxicology studies using zebrafish embryos as the model organism. Our findings reveal that these emerging 2,6-DHNPs showed lethal toxicity 248 times greater than that of the regulated DBP, dichloroacetic acid. Specifically, at sublethal concentrations, exposure to 2,6-DHNPs generated reactive oxygen species (ROS), caused apoptosis, inhibited cardiac looping, and induced cardiac failure in zebrafish. Remarkably, the use of a ROS scavenger, N-acetyl-l-cysteine, considerably mitigated these adverse effects, emphasizing the essential role of ROS in 2,6-DHNP-induced cardiotoxicity. Our findings highlight the cardiotoxic potential of 2,6-DHNPs in drinking water even at low concentrations of 19 μg/L and the beneficial effect of N-acetyl-l-cysteine in alleviating the 2,6-DHNP-induced cardiotoxicity. This study underscores the urgent need for increased scrutiny of these emerging compounds in public health discussions.

## Highlights

• 2,6-DCNP and 2,6-DBNP exhibited considerable resistance to removals in drinking water treatment plants.
• The levels of 2,6-DCNP and 2,6-DBNP showed the most significant reduction (47% and 52%) in boiling and filtration.
• 2,6-DHNP exposures caused heart failure via mediating ROS and delaying heart development.
• N-acetyl-L-cysteine mitigated 2,6-DHNP-induced cardiotoxicity.

## Graphical abstract

**Figure:**
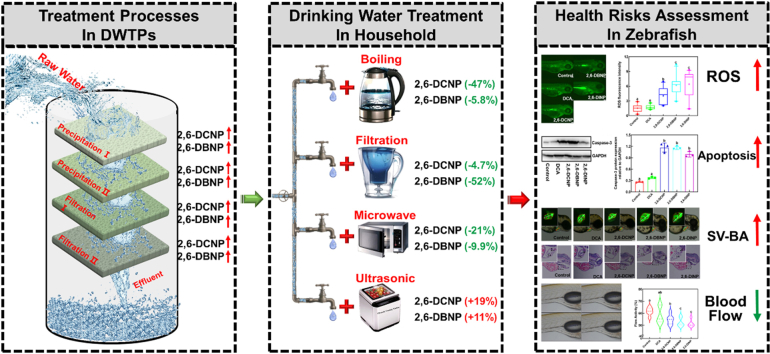

## 1 Introduction

The occurrence of disinfection byproducts (DBPs) in water is an unintended consequence of water disinfection [1]. Over 800 DBPs have been identified in water, among which trihalomethanes and haloacetic acids are classified as regulated DBPs due to their known risk to humans [2]. Recently, some emerging aromatic halogenated DBPs have been frequently detected in water, attracting much attention because of their higher toxic potencies compared to regulated DBPs [3,4]. These aromatic halogenated DBPs are divided into four categories according to their chemical structures, i.e., trihalogenated phenols, dihalogenated nitrophenols (2,6-DHNPs), dihalogenated hydroxybenzoic acids, and dihalogenated hydroxybenzaldehydes [5]. Among these four categories, 2,6-DHNPs showed higher toxic potencies, exhibiting 101% more developmental toxicity than corresponding trihalogenated phenols in the marine polychaete *Platynereis dumerilii*, and 32-fold more cytotoxic than 3,5-dichloro-4-hydroxybenzaldehyde and 3,5-dichloro-4-hydroxy-benzoic acid in Chinese hamster ovary cells [6,7]. Furthermore, 2,6-DHNPs are more persistent and more difficult to be photolyzed than other aromatic halogenated DBPs due to the presence of nitro groups in 2,6-DHNPs, which facilitates the establishment of intramolecular hydrogen bonds and thus increases their public health risks [[8], [9], [10]].

At present, some studies have detected 2,6-DHNPs in various water samples, such as sewage effluent, swimming pool water, and drinking water [7,11,12]. The ubiquitous 2,6-DHNPs further raise public concerns about their health risks. Some studies have demonstrated that 2,6-DHNPs showed relatively high developmental toxicity to *P. dumerilii* [6], comparatively high cytotoxicity to HepG2 cells [8], and relatively high binding affinities with human transthyretin and catalase [7,13]. However, this information is insufficient to understand the health risks of 2,6-DHNPs, a group of high toxic potency DBPs that are ingested daily by humans via drinking water. Therefore, further investigations are needed to better understand their potential health risks.

Household water treatment (HWT) plays a crucial role in determining the ultimate levels of DBPs that enter the human body, serving as the final defense to ensure drinking water safety [14]. Neglecting to account for household treatment of drinking water can exaggerate the estimations of public health risks associated with DBPs in drinking water. Previous studies have demonstrated the effectiveness of HWT in removing more than 60% of regulated DBP, such as trihalomethanes and haloacetic acids, from tap water [15]. However, no information is available regarding the impact of HWT on emerging DBP 2,6-DHNPs. The enhanced electrophilic reactivity and greater stability exhibited by 2,6-DHNPs relative to trihalomethanes and haloacetic acids introduce unpredictable consequences, thereby underscoring the need to investigate the removal capacities of HWT, specifically towards 2,6-DHNPs before assessing their associated health risks.

Zebrafish (*Danio rerio*) embryos have a comprehensive multicellular system that can effectively model integrated physiological processes. Moreover, their transparency makes them ideal for noninvasive and whole-animal imaging. With the added benefits of higher genome similarity with humans, swift ex-utero development, and high fecundity, zebrafish embryos stand out as exemplary model organisms [16,17]. Herein, the zebrafish embryo was employed to explore the adverse health effects of 2,6-DHNPs on humans. Since heart is the first form and functional organ, it is more susceptible to pollutant exposures compared to other organs [18]. Therefore, the impacts of 2,6-DHNPs on the cardiac impacts of zebrafish larvae were also assessed. Overall, the objectives of this study are to: (1) explore the occurrence of 2,6-DHNPs in the water samples from drinking water treatment plants (DWTPs) and tap, follow the removal efficiencies of HWT on 2,6-DHNPs (Fig. S1); (2) assess the adverse health risks of 2,6-DHNPs on the zebrafish embryo by determining the median lethal concentration (LC_50_) and sublethal concentration (SC; as 10% LC_50_) and evaluating various indicators; and (3) examine the effects of 2,6-DHNPs on zebrafish cardiac development and function as well as the underlying mechanism of 2,6-DHNP-induced cardiotoxicity such as the role of reactive oxygen species (ROS). In summary, this study endeavors to shed light on the health implications of 2,6-DHNPs in zebrafish, offering insights into their risk for humans and broader public health.

## 2 Materials and method

### 2.1 Sampling of drinking water

Water samples were collected from two DWTPs (A and B) and Zhejiang Normal University (ZJNU) in Jinhua, China. DWTP A sourced its water from the Shafan reservoir, while DWTP B sourced its water from the Andi reservoir, with their treatment capacities at 0.3 and 0.5 million m^3^/day, respectively. The water samples from the DWTPs were collected after undergoing chlorination, sedimentation, and filtration. The water samples from ZJNU were collected on April 13, 2023, from a faucet in Building 8 that provides daily drinking water for about 400 adults. Prior to sample collection, these faucets were allowed to run for 5 min to flush out residual stagnant water. Water samples were collected in 1 L pure glass bottles and sealed with polypropylene caps and silicone septa.

The residual chlorine was quantified using the N,N-diethyl-p-phenylenediamine (DPD) titrimetric method. To avoid further DBP formation, a 120% stoichiometric amount of ascorbic acid (0.28 mol/L) was added into the sample immediately. All samples were filtered with 0.45 μm glass fiber filters and then stored at 4 °C in the refrigerator until analyses. In addition, water quality parameters, including dissolved organic carbon, absorbance at 254 nm (UV_254_), specific ultraviolet absorbance (SUVA), pH, salinity, conductivity, total dissolved solids, bromide (Br^−^), iodide (I^−^), total nitrogen, ammonia nitrogen (NH_3_–N), nitrate nitrogen (NO_3_–N), and nitrite nitrogen (NO_2_–N) were measured (Table S3).

### 2.2 Household water treatments

HWTs are widely promoted as appropriate interventions to improve drinking water safety [19]. Four typical HWTs, including boiling, filtration, microwave, and ultrasound, have been proven to reduce regulated DBPs [20,21], and thus were evaluated for the removal effectiveness on 2,6-DHNP levels in tap water herein (Fig. S3).

Boiling, particularly prevalent in Asian countries, is effective in reducing DBP concentrations in drinking water [14]. In this study, an electric kettle (WSJ1703b, Midea, China) equipped with an automatic shut-off feature was utilized to heat tap water. A volume of 500 mL of tap water was added to the kettle before the heating process commenced. The heat source was promptly disabled upon reaching the boiling point of water. Subsequently, the boiled water was allowed to cool down to ambient temperature and subjected to extraction and analysis as per the established protocol.

In-house filtration, a convenient approach to enhance drinking water quality, has gained popularity in households [22]. In this study, a filter bottle (Marella Marine Series 3.5L, Brita, China) equipped with an activated carbon adsorption filter cartridge was employed to purify tap water. Prior to use, the filter cartridge was cleansed with 5 L of water to eliminate any impurities. Subsequently, the tap water was passed through the filter bottle, wherein the activated carbon filter cartridge effectively removed the contaminants. 500 mL of filtered water was carefully collected for subsequent extraction and analysis.

Microwave ovens, common for heating food and soup, represent a prevalent point of interaction between tap water and the general public [23]. In the study, a microwave oven (P70OF20CL-DG, Galanz, China) was utilized to heat the tap water. A 250 mL porcelain tank with a lip was placed in the microwave oven. The tap water underwent microwave irradiation for 4 min, reaching a final temperature of 95.5 °C. After each treatment cycle, the porcelain tank containing the heated water was carefully removed from the microwave oven and allowed to cool down to room temperature. This process was repeated until 500 mL of water was collected. Subsequently, the collected water was prepared following the established extraction and analysis protocol.

Ultrasonic cleaners, used for cleaning various foods (especially vegetables and fruits), may play a role in safeguarding public health through their potential for removing contaminants [24]. In the study, a 30 W ultrasonic cleaner (KQ2200, Kelong, China) was employed to ultrasound the tap water. A glass beaker containing 500 mL of tap water was carefully placed inside the ultrasonic cleaner for a 40-min ultrasonic treatment. Following the completion of the ultrasonic treatment, the tap water was prepared for subsequent extraction and analysis following the established protocol.

### 2.3 Cardiac development toxicities of 2,6-DHNPs and DCA using transgenic zebrafish

Tg (*cmlc*: EGFP) zebrafish, which specifically expresses the enhanced green fluorescent protein (EGFP) in myocardial cells, was used to examine the changes in the distance between sinus venosus (SV) and bulbus arteriosus (BA) to indicate the cardiac development toxicity [25]. In this study, the adverse effects of 2,6-DHNPs on cardiomyogenesis were performed. Briefly, 10 larvae of Tg (*cmlc*: EGFP) zebrafish in each treatment were randomly selected and anesthetized (0.168 mg/mL MS-222) for 1 min. Subsequently, the distance from sinus venosus to bulubs arteriosus (μm) was measured at 72 hpf using a fluorescent microscope (BX43, Olympus, Japan) and quantified by Image J (Bethesda, MD).

### 2.4 Apoptosis using acridine orange staining

In the study, acridine orange staining was used [26]. Briefly, after exposure to 2,6-DHNPs for 72 h, 15 larvae of each treatment were stained with acridine orange solution (2 mg/L in an E3 solution) in darkness for 30 min. After washing with E3 solution for 5 min, these larvae were anesthetized with 0.03% MS-222 for 3 min. Apoptotic cells were visualized using a fluorescence microscope (BX43, Olympus, Japan), and the fluorescence intensity of individual larvae, determined by the area of integrated optical density, was quantified using ImageJ software.

### 2.5 Measurement of ROS and N-acetyl-l-cysteine

ROS generation was determined to understand the health effect mechanism of 2,6-DHNPs in zebrafish larvae. Briefly, the larvae were incubated in the dark for 1 h with 20 μM DCFH-DA at 28 °C. After anesthetized with 168 mg/L MS-222 for 1 min, the ROS levels in these larvae were evaluated using the fluorescence microscope (BX43, Olympus, Japan). The fluorescence intensity of ROS staining was calculated using Image J (Bethesda, USA). N-acetyl-l-cysteine (NAC), a ROS scavenger, was used to protect zebrafish from ROS-induced effects in this study. To determine the optimal concentrations of NAC, preliminary experiments were conducted. In the experiments, 0, 50, and 100 μΜ NAC were tested to eliminate ROS. After exposure for 72 h, we found that 50 μM NAC was the most effective in eliminating ROS among the three doses of DBP (Fig. S2). Therefore, 50 μM NAC was used as the antioxidative component to eliminate the effect of ROS in the following experiments.

### 2.6 Statistical analysis and quality control

All figures were drafted by GraphPad Prism 9 and Origin 2022. All statistical analyses were performed using SPSS 25.0. Differences were determined by a one-way analysis of variance followed by Duncan's multiple-range test. Differences were considered significant when *p* < 0.05. During the static tests, the recovery rates (measured concentrations of the test substances as a percentage of the nominal concentrations) of the test solutions ranged from 95% to 106%, indicating stable and constant exposure doses in this study. The detected ranges of 2,6-DHNPs for the nominal concentrations (0.034, 0.032, and 0.019 mg/L) were 0.0323, 0.0313, and 0.0201 mg/L, respectively.

## 3 Results and discussions

### 3.1 The occurrence of 2,6-DHNPs in DWTPs and tap water

Previous studies have identified 2,6-DHNPs (2,6-dichloro-4nitrophenol [2,6-DCNP], 2,6-dibromo-4-nitrophenol [2,6-DBNP], and 2,6-diiodo-4-nitrophenol [2,6-DINP]) as a group of emerging aromatic DBPs frequently detected in water environments [5,11]. To verify the persistence of 2,6-DHNPs against water treatment approaches, two DWTPs in Jinhua, China were sampled to determine 2,6-DHNPs at each consecutive stage of the drinking water treatment process. As shown in Table S2, 2,6-DCNP and 2,6-DBNP were found in the influent water of DWTPs, which could be attributed to the use of phenolic pesticides in agricultural production in the surrounding farmland [27]. Previous research confirms the widespread use of DHNPs in agricultural and industrial chemicals [27,28]. The water treatment process in the DWTP, encompassing chlorination, coagulation, and filtration stages, was designed to convert influent water into potable water by removing impurities. However, the levels of 2,6-DCNP and 2,6-DBNP were increased after chlorination and were not eliminated during subsequent coagulation and filtration stages. The increases in 2,6-DCNP and 2,6-DBNP were likely due to the phenol compounds transformed into DHNPs during chlorination process [29]. Similarly, Yang and Zhang [6] have detected 2,6-DCNP and 2,6-DBNP in sewage treatment effluents. Their persistence during treatment processes suggests that 2,6-DHNPs may form during chlorination of DWTP, and conventional drinking water treatment is incapable of eliminating these compounds effectively. This phenomenon could be attributed to the stable physicochemical properties of 2,6-DHNPs, such as the greater electron-withdrawing ability of the nitro group of chemicals [9]. Additionally, the presence of 2,6-DINP was not detected at any stage of DWTPs, likely owing to the exceedingly low iodine levels in influent water (Table S3).

In short, 2,6-DCNP and 2,6-DBNP are frequently detected in influent water, and their concentrations often increase following chlorination at DWTP. These compounds exhibit considerable resistance to removal during coagulation, precipitation, and filtration stages, thus resulting in household tap water containing 2,6-DHNPs.

### 3.2 The impacts of HWTs on 2,6-DHNP levels in drinking water

HWTs augment existing strategies for DBP treatment, showing significant potential to lower DBP levels in drinking water [19]. To better understand the persistence of 2,6-DHNPs and justify the significance of their toxicity assessments, this study scrutinizes the influence of four prevalent HWTs (boiling, filtration, microwave irradiation, and ultrasonic cleaning) on the concentrations of 2,6-DHNPs in tap water as a prior step toward assessing their potential health risks. In this study, we found that four HWTs have significant effects on 2,6-DCNP and 2,6-DBNP levels in tap water (Table 1). Of these HWTs, boiling, filtration, and microwave irradiation significantly decreased 2,6-DCNP and 2,6-DBNP levels, with the decrease of 47%, 4.7%, and 20% for 2,6-DCNP levels, and 6.0%, 52%, and 9.9% for 2,6-DBNP levels, respectively. The decline of 2,6-DCNP and 2,6-DBNP in boiling can be attributed to decarboxylation and dehalogenation processes during boiling [15]. However, the reduction in 2,6-DBNP was notably less than that of 2,6-DCNP in boiling. This mirrors the findings by Pan et al. noting a higher rate of volatilization for brominated DBPs than their chlorinated counterparts, attributed to their lower boiling points and increased volatility of the latter [21]. The contrasting impact of filtration versus boiling on these compounds is likely due to differences in their aqueous solubility and polarity. Similarly, Weinberg et al. [30] found that bromine-containing congeners have greater filtration removal efficiency than trichloromethane, dichloroacetic acid (DBP), and trichloroacetic acid due to their lesser solubility and polarity. Whereas the differential removal outcomes from microwave heating and boiling may be attributed to the distinct heating mechanisms involved. Unlike boiling, microwaves heat water via irradiation from the sides, inciting advanced oxidation reactions that cleave chemical bonds and transform large molecules in 2,6-DCNP.

**Table 1:** Impacts of four common household water treatments (filtration, boiling, microwave, and ultrasonic) on the 2,6-DHNP levels in tap water.

Sample site	Treatment	2,6-DCNP (ng/L)	2,6-DBNP (ng/L)	2,6-DINP (ng/L)
Tap water	Control	2.88 ± 0.04^a^	2.16 ± 0.06^a^	ND
	Boiling	1.53 ± 0.04^c^	2.03 ± 0.07^c^	ND
	Filtration	2.75 ± 0.07^b^	1.03 ± 0.05^b^	ND
	Microwave	2.29 ± 0.05^d^	1.95 ± 0.02^d^	ND
	Ultrasonic	3.42 ± 0.08^e^	2.39 ± 0.08^e^	ND
Tap water (add 50 μg/L 2,6-DHNPs standards)	Control	43.01 ± 0.49^a^	45.72 ± 0.10^a^	43.89 ± 0.40^a^
	Boiling	21.23 ± 0.06^b^	43.27 ± 0.21^c^	38.90 ± 0.58^c^
	Filtration	44.24 ± 0.04^a^	23.55 ± 0.05^b^	23.39 ± 0.18^b^
	Microwave	39.95 ± 0.28^c^	35.84 ± 0.83^d^	33.51 ± 0.19^d^
	Ultrasonic	49.38 ± 0.58^d^	46.83 ± 0.05^a^	46.99 ± 0.22^e^

Different letters indicate significant differences (*p* < 0.05). 2,6-DHNPs, dihalogenated nitrophenols; 2,6-DCNP, 2,6-dichloro-4nitrophenol; 2,6-DBNP, 2,6-dibromo-4-nitrophenol; 2,6-DINP, 2,6-dibromo-4-nitrophenol; ND, not detected.

In contrast, ultrasonic cleaning showed opposite effect, which significantly increased 2,6-DCNP and 2,6-DBNP levels, increasing by 18% and 10% in 2,6-DCNP and 2,6-DBNP treatments, respectively. These increases may be ascribed to transformations of large molecules under ultrasonic conditions, aligning with previous research indicating the limited effectiveness of ultrasonic devices on the removal of chloral hydrate [31].

In addition, another experiment was conducted to validate 2,6-DHNP removal efficiencies during the tested HWTs (Table 1). Tap water was spiked with 50 μg/L of 2,6-DHNPs and treated using the four household methods (Fig. S4). The results were consistent with our HWT data, thus reaffirming both the reliability of our HWT procedures and the accuracy of our analyses.

In short, among the four common HWTs, boiling and filtration showcased the best reduction efficacy of 47% and 52% for 2,6-DCNP and 2,6-DBNP, respectively. These findings emphasize the inevitable human consumption of persistent 2,6-DHNPs, underscoring significant concerns regarding their potential risks to public health. Consequently, it becomes imperative to undertake comprehensive health risk assessments regarding these emerging and persistent contaminants.

### 3.3 Health risk assessments of 2,6-DHNPs using zebrafish embryo

Given their resistance to HWT procedures, the ubiquity of 2,6-DHNPs in drinking water poses public health risks via inevitable human exposures. Zebrafish embryo is an exceptional model for assessing the potential human health risks posed by hazardous chemicals [32], which was utilized to evaluate the adverse health effects of 2,6-DHNPs on humans.

**Fig. 1:**
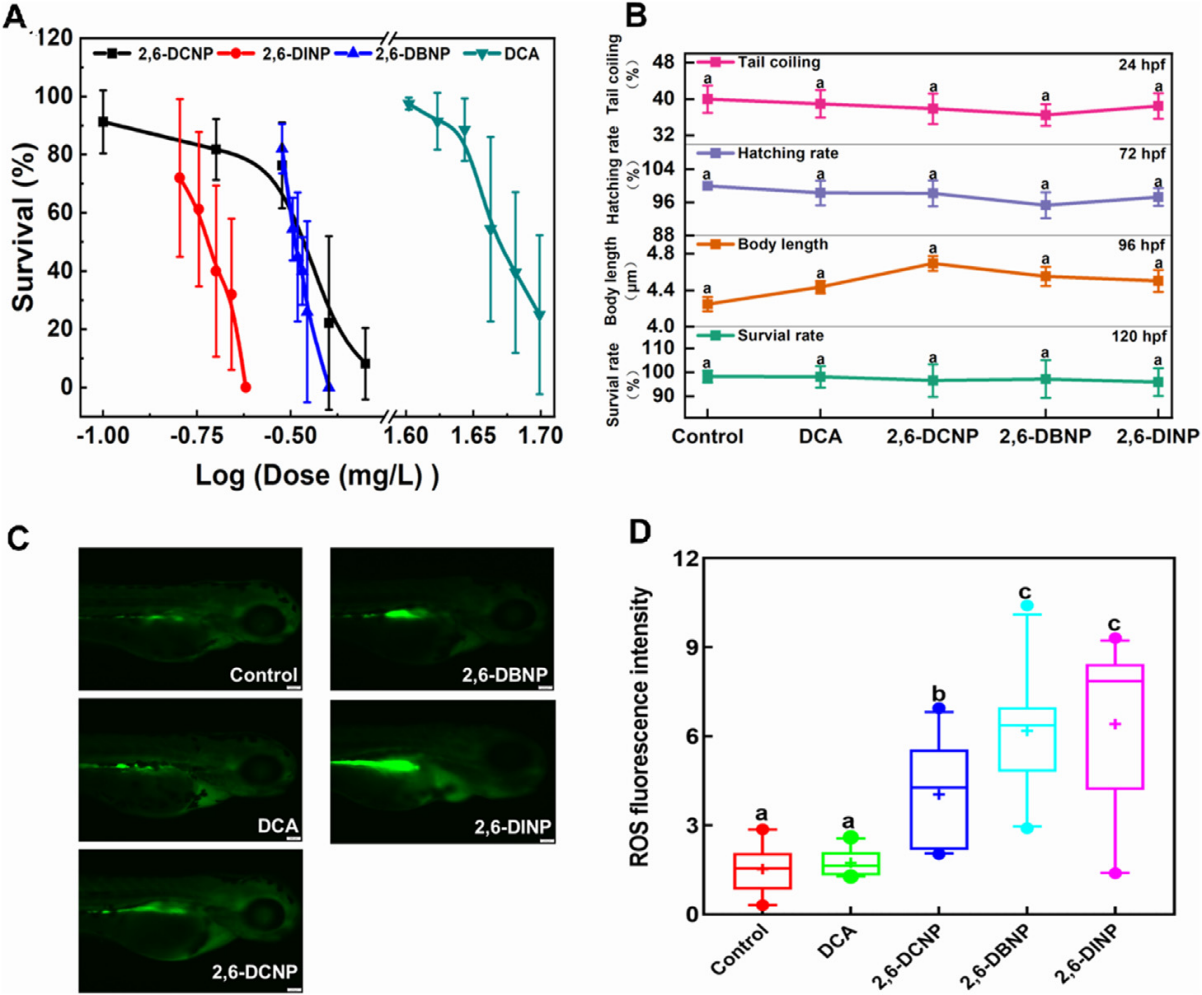
Impacts of 2,6-DHNPs and DCA on the early development of zebrafish larvae. (A) Survival rate at 120 hpf (n = 6); (B) Spontaneous tail coiling of 24 hpf, hatching rate of 72 hpf, body length of 96 hpf, and survival rate of 120 hpf at SCs; (C) Distribution of fluorescence visualizing ROS in zebrafish larvae; and (D) Fluorescence intensity of ROS. Boxes represent the 5th and 95th percentiles, the error bar represents the 1st and 99th percentiles, and the line in the box represents the mean value. Different letters denote significant differences at *p* < 0.05 (n = 10). ROS, reactive oxygen species; SC, sublethal concentration; DCA, dichloroacetic acid.

In this study, the detected concentrations of 2,6-DCNP, 2,6-DBNP, and 2,6-DINP were 0.0323, 0.0313, and 0.0201 mg/L, respectively. These detected concentrations were less than 20% deviations from expected concentrations (0.034, 0.032, and 0.019 mg/L), implying that the expected concentrations can represent the actual content in this work. As expected, the survival rates of zebrafish larvae were negatively correlated with DHNP concentrations (Fig. 1A). Based on their dose–response curves, the 120 h-LC_50_ values of 2,6-DCNP, 2,6-DBNP, 2,6-DINP, and DCA were 0.34, 0.32, 0.19, and 47.1 mg/L, respectively (Fig. 1A). The findings suggest that 2,6-DHNPs exhibited toxicity levels up to 248 times higher than the regulated DCA in zebrafish larvae, which aligns with a previous study demonstrating that 2,6-DHNPs exerted developmental toxicity levels 165 times greater than the regulated DBP in marine polychaete *P. dumerilii* [6]. Like other halogenated organic compounds, 2,6-DHNPs show expected toxicity ranking as 2,6-DINP > 2,6-DBNP ≈ 2,6-DCNP. This is likely due to the higher electrostatic potential of iodine, which consequently leads to greater toxicity than that of bromine and chlorine [33].

Alterations in specific early life-stage endpoints of zebrafish, such as survival rate, body length, hatchability, and spontaneous tail coiling, often arise from environmental influences and are deemed early-warning indicators for assessing the toxicological risks (e.g., survival, growth, development, and early behavior) associated with environmental pollutants [34,35]. In this study, we demonstrated that 2,6-DHNPs at SC did not show significant effects on spontaneous tail coiling at 24 hpf, hatchability at 72 hpf, body length at 96 hpf, and survival rate at 120 hpf (Fig. 1B). These results suggest that at SCs, 2,6-DHNPs appear to be safe, showing no obvious effect on the survival, growth, development, and early behavior of zebrafish larvae.

Further, reactive oxygen species (ROS) is a vital factor in DBPs-induced toxicities [36]. Thus, the levels of ROS were assessed after 2,6-DHNPs exposure. The results reveal that ROS levels did not significantly change under DCA exposure, while significantly increased under 2,6-DHNP exposure at 72 hpf (Fig. 1C and D). This data suggests that the regulated DCA could not generate ROS at SCs, while 2,6-DHNPs have the potential to induce ROS generation even at SCs, indicating 2,6-DHNPs as potent ROS inducers. Our result was consistent with other emerging DBPs, such as 2,6-dichlorobenquinone, which generate ROS in zebrafish larvae [26]. These results indicate that 2,6-DHNP exposures can disrupt the oxidation balance in zebrafish.

In short, our study is the first to reveal the lethal toxicity of 2,6-DHNPs in zebrafish larvae. Furthermore, 2,6-DHNPs are potent ROS inducers that can generate ROS even at SCs, thus posing human health risks and underscoring their potential threat to public health.

### 3.4 The impacts of ROS induced by 2,6-DHNP exposures

**Fig. 2:**
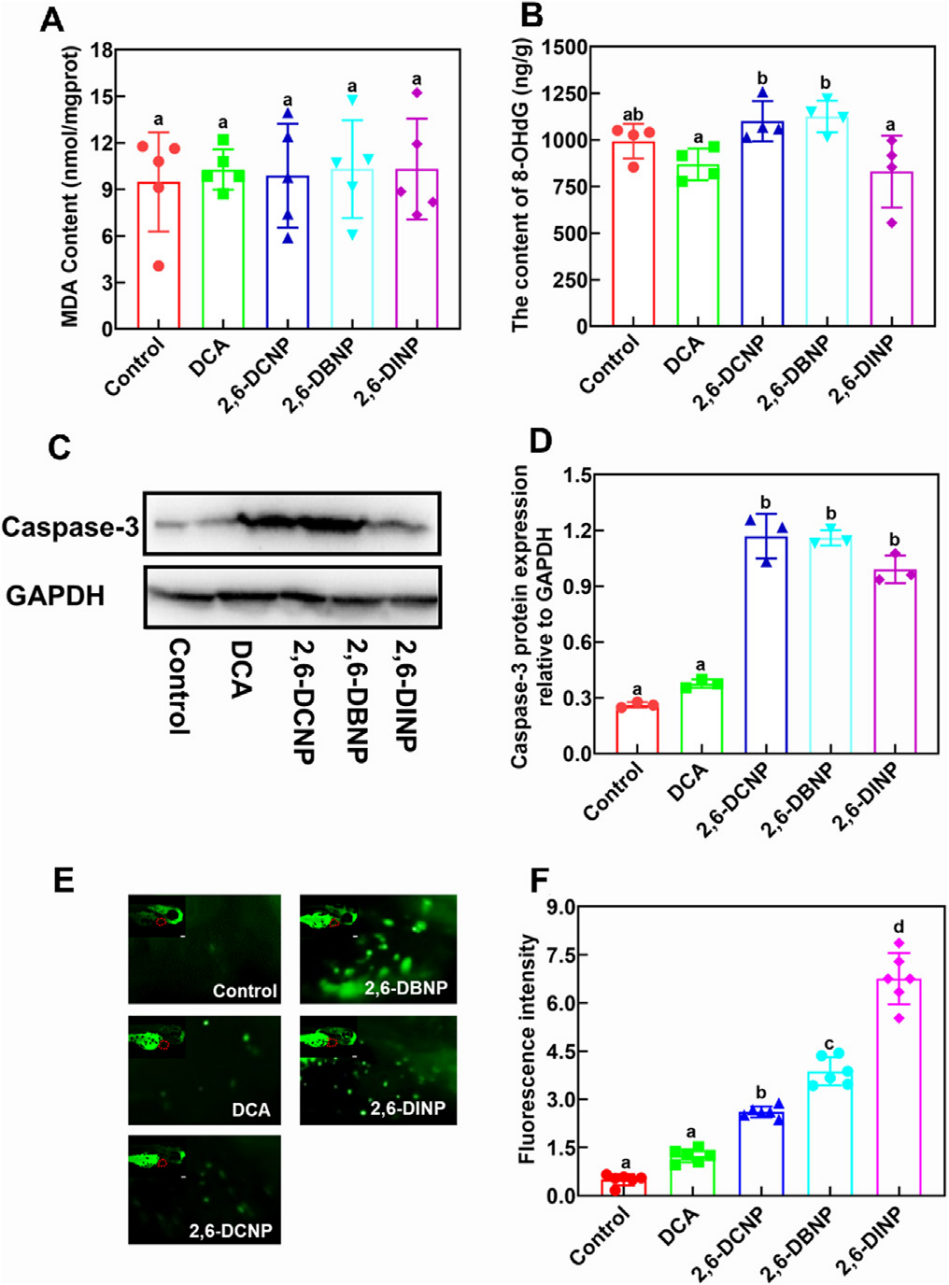
Impacts of 2,6-DHNPs and DCA on MDA contents (A), 8-OHdG contents (B), the protein expression levels of caspase-3 (C and D), and apoptosis performance (E and F). (C) Caspase-3 and GAPDH protein expressions determined by western blotting in control and 2,6-DHNP treatments; (D) Caspase-3 protein levels were quantified by densitometry; (E) AO staining of zebrafish larvae; (F) Fluorescence intensity of AO staining. Vertical bars represent ±SD, and different letters above bars indicate significant differences at *p* < 0.05. MDA, malonaldehyde; GAPDH: glyceraldehyde 3-phosphate dehydroge nase; AO, acridine orange.

ROS is highly active and can induce various toxicities by reacting indiscriminately with cellular components such as DNA, proteins, and lipids [37]. Malonaldehyde (MDA) and 8-hydroxydeoxyguanosine (8-OHdG) serve as biomarkers for evaluating oxidative damage to cell membranes and DNA caused by ROS [38,39]. In this study, 2,6-DHNP exposures did not have a significant effect on MDA and 8-OHdG levels (Fig. 2A and B), indicating that ROS generated by 2,6-DHNPs at SCs cannot cause damage to cell membranes and DNA. However, 2,6-DHNPs can induce apoptosis by triggering caspase-3, a critical apoptotic-related protein [40]. We found that caspase-3 expressions were significantly enhanced by ∼4-fold after 2,6-DHNP exposures (Fig. 2C and D), indicating that 2,6-DHNPs can induce apoptosis at SCs. Combined with the results of MDA and 8-OHdG, 2,6-DHNPs are more capable of elevating caspase-3 expression and inducing apoptosis compared with damaging the DNA and lipids of membranes.

To investigate the impact of 2,6-DHNPs on apoptotic performance, we used acridine orange to mark the sites of apoptosis in zebrafish [41]. As a result, exposure to 2,6-DHNPs significantly increased fluorescence intensity, indicating strong apoptosis effects, with major apoptotic cells distributing in the heart area of zebrafish larvae (Fig. 2E and F). The data suggest that 2,6-DHNP exposures primarily caused apoptosis in the heart of zebrafish larvae. Similarly, 2,6-DHNPs also exhibited different capacities in inducing apoptosis when compared with control, in the order of 2,6-DINP (13.5-fold) > 2,6-DBNP (7.1-fold) > 2,6-DCNP (5.2-fold), attributed to their different nucleophilicities similar to their toxicity ranking.

Collectively, ROS induced by 2,6-DHNP exposure poses a non-negligible threat to the early life-stage of zebrafish by generating ROS and inducing apoptosis. Further, the occurrence of major apoptosis observed in the heart indicates that 2,6-DHNP exposures could disrupt the normal heart development of zebrafish.

### 3.5 The impacts of 2,6-DHNP exposures on the cardiac development of zebrafish embryos

The zebrafish embryo heart matures and becomes functional within a mere 72 h, rendering it particularly vulnerable to environmental contaminants due to the complex interplay of cellular proliferation, migration, differentiation, and intricate morphogenetic interactions throughout the cardiogenic process. Consequently, even subtle disturbances can compromise normal cardiac development in zebrafish larvae [42,43]. Herein, the impacts of 2,6-DHNP exposures on cardiac development were assessed using Tg (*cmlc*: EGFP) zebrafish larvae. The looping process, a pivotal stage in early cardiac morphogenesis, involves the gradual bending of the linear heart tube at the boundary between the sinus venosus (SV) and the bulbus arteriosus (BA), resulting in an S-shaped loop. Therefore, the total distance between SV and BA in the Tg (*cmlc*: EGFP) zebrafish larvae was employed as an indicator of cardiac development [44].

**Fig. 3:**
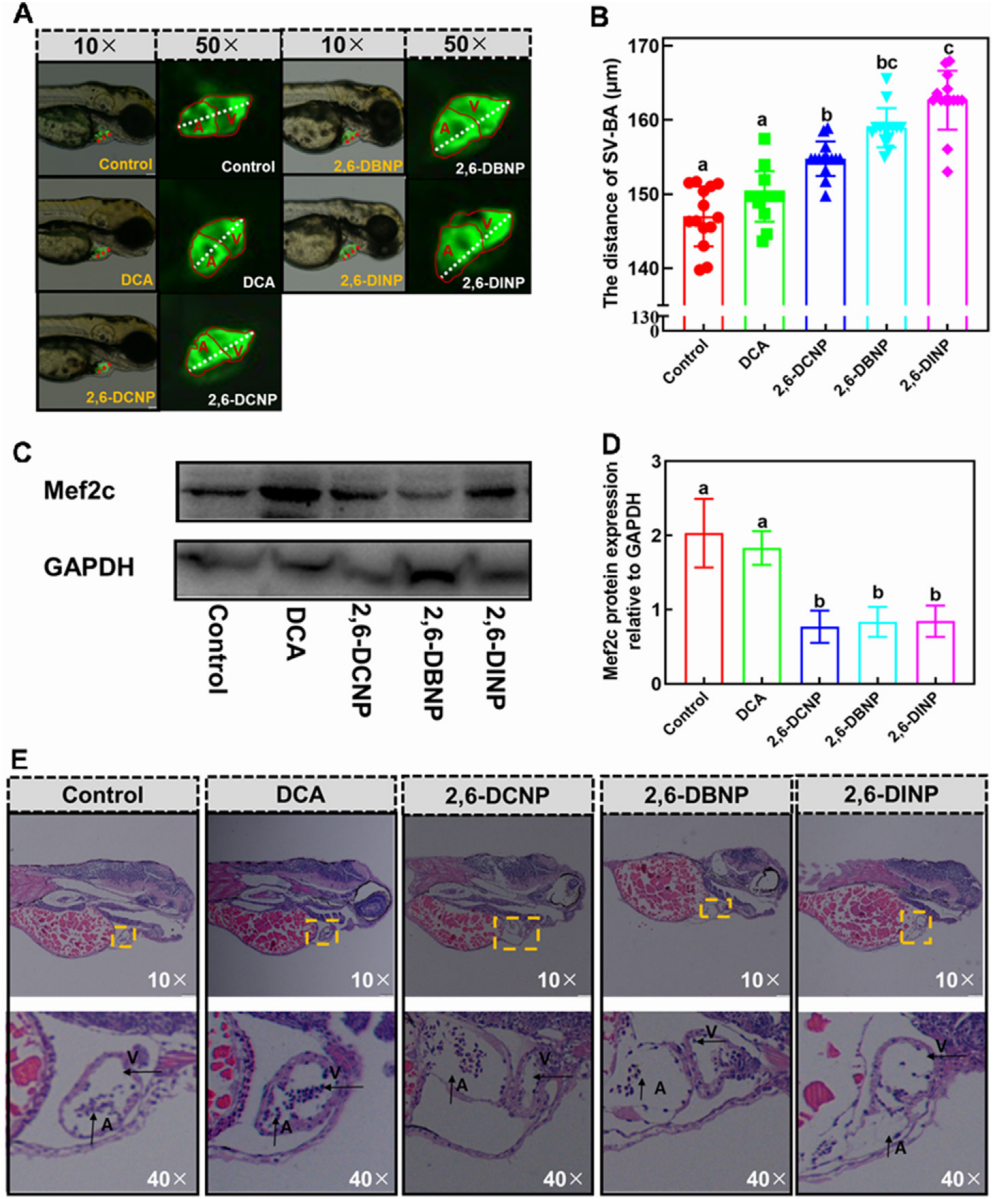
Impacts of 2,6-DHNPs and DCA on the SV–BA distances in Tg (*cmlc*: EGFP) zebrafish larvae (A and B), Mef2c expressions (C and D), and histopathological changes of the heart (E) in zebrafish larvae. (A) The merging images of Tg (*cmlc*: EGFP) zebrafish larvae in bright and fluorescence fields in control and 2,6-DHNP treatments; (B) the distance of SV-BA in Tg (*cmlc*: EGFP) zebrafish larvae in control and 2,6-DHNP treatments; (C) Mef2c and GAPDH protein expressions determined by western blotting in control and 2,6-DHNP treatments; (D) Mef2c protein levels were quantified by densitometry; (E) the histopathological photos of heart in normal zebrafish larvae in control and 2,6-DHNP treatments; Vertical bars represent ±SD, and different letters above bars indicate significant differences at *p* < 0.05. EGFP, enhanced green fluorescent protein; SV–BA, sinus venosus–bulbus arteriosus.

In this study, the hearts in the control group developed well, displaying two largely overlapped chambers. However, exposure to 2,6-DHNPs increased the distance between SV and BA, resulting in a diminished overlap area in the heart of Tg (*cmlc*: EGFP) zebrafish larvae (Fig. 3A and B) [45,46]. This increased SV–BA distance suggests that the zebrafish larval heart failed to undergo proper looping, becoming stretched and elongated under 2,6-DHNP exposures. This indicated that 2,6-DHNP exposure delayed cardiac development, leading to heart enlargement in zebrafish larvae. Mef2c, a crucial cardiomyogenic regulator expressed in heart precursor cells, orchestrates cardiac morphogenesis, particularly linear heart tube formation and right ventricular development [47,48]. Correspondingly, subsequent western blotting of Mef2c revealed a significant 38%, 41%, and 42% decrease in expression under 2,6-DCNP, 2,6-DBNP, and 2,6-DINP exposures, respectively (Fig. 3C and D). This reduction in Mef2c protein expression suggested that 2,6-DHNP exposures hindered cardiac looping in zebrafish, aligning with previous findings that Mef2c deficiency induced cardiac looping defects in mice [49].

In addition, the impacts of 2,6-DHNP exposures on cardiac structure were verified by histopathological experiments. We found that 2,6-DHNP exposures caused changes in looping, compaction of the ventricle, elongation of the atrium, and shrinking of the luminal area (Fig. 3E). Such outcomes indicate that early life-stage exposure to 2,6-DHNPs can delay the looping of the heart tube into a distinctive two-chambered structure. This is consistent with previous research showing that 2,3,7,8-tetrachlorodibenzo-p-dioxin disrupted cardiac development via augmenting SV–BA distances in zebrafish larvae [50], thus suggesting that 2,6-DHNPs could disrupt cardiac development.

In short, our findings indicate that 2,6-DHNP exposures inhibit zebrafish larval cardiac looping, thereby hindering cardiac development. Given the intricate cellular and molecular processes required to form a mature, blood-pumping organ during zebrafish embryonic development, chemical stressors like 2,6-DHNPs could disrupt cardiac development. Therefore, a comprehensive investigation is crucial to fully understand the mechanisms underlying 2,6-DHNP-induced cardiac developmental toxicity.

### 3.6 Impacts of 2,6-DHNPs on gene expressions related to cardiac development in zebrafish larvae

**Fig. 4:**
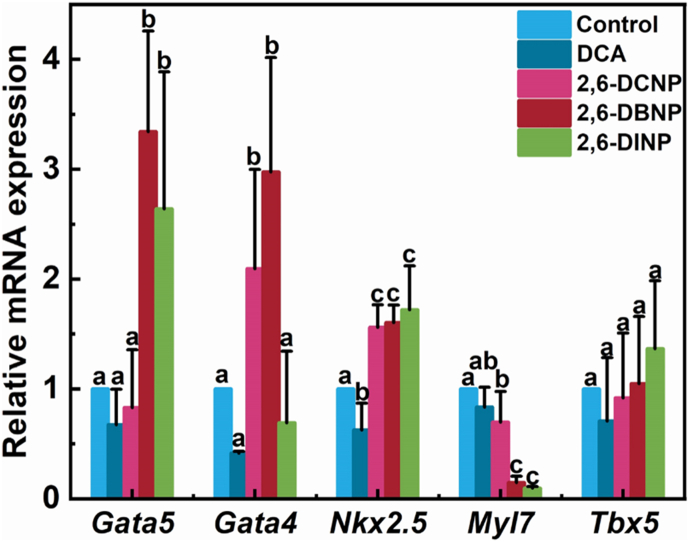
Impacts of 2,6-DHNPs and DCA on the gene (*Gata5*, *Gata4*, *Nkx2.5*, *Myl7*, and *Tbx5*) mRNA transcript expression levels. Vertical bars represent ±SD, and different letters above bars indicate significant differences at *p* < 0.05.

Cardiogenesis, the formation of the chambered heart, is a highly complex process involving specification, differentiation, migration, and maturation [51]. During cardiogenesis, a series of transcription factors are required to switch on and off in specific temporal and spatial patterns to orchestrate the key anatomical and functional processes leading to cardiac formation [52,53]. Some key evolutionarily conserved transcription factors (*Gata5, Gata4, Nkx2.5, Cmlc,* and *Tbx5*) were assayed to further explore the impacts of 2,6-DHNPs on cardiac development at molecular levels. Among these transcription factors, *Gata5* is responsible for producing normal numbers of myocardial precursors in zebrafish [54]. As shown in Fig. 4, the *Gata5* mRNA transcriptional levels show no obvious change under 2,6-DCNP exposure, but were significantly elevated to 234% and 164% under 2,6-DBNP and 2,6-DINP exposures, respectively. This result indicates that 2,6-DBNP and 2,6-DINP exposures induced cardiac myocyte production in zebrafish larvae. Considering the previously mentioned results on apoptosis and SV–BA distance, it was surmised that the lack of *Gata5* mRNA transcription alteration in 2,6-DCNP might be because the limited increase in apoptosis and SV–BA distance induced by 2,6-DCNP was insufficient to activate a molecular-level *Gata5* response. Conversely, the increased *Gata5* mRNA transcription levels after 2,6-DBNP and 2,6-DINP exposures may have contributed to cardiomegaly, necessitating greater cardiac myocyte involvement. The result reported herein was consistent with the previous study [55], which also indicates that the overexpression of *Gata5* leads to enlarged hearts in zebrafish.

Unlike *Gata5*, *Gata4* is an early marker of the cardiac cells, which play crucial roles in heart specification and development [56]. In this study, the *Gata4* transcript level showed no apparent change with 2,6-DINP treatment, but was significantly elevated with increases of 109% and 197% under 2,6-DCNP and 2,6-DBNP exposures (Fig. 4), implicating that 2,6-DHNP exposures can disturb the normal cardiac development of zebrafish embryo. A possible reason for this increased expression may be attributed to the induced cardiomegaly, which needs to recruit more cells to the cardiogenic field. However, 2,6-DINP exposure, which exerts the highest toxic effect by generating the most ROS, induces the most apoptosis, and enhances the most SV–BA distance, has exceeded *Gata4* regulatory capacity [57]. A similar case was also reported by Liang et al., who demonstrated that the increase of *Gata4* transcription can induce hypertrophic responses in cardiac myocytes, either by *in vivo* or *in vitro* assays [58].

NK2 transcription factor related 5 (*Nkx2.5*) is a critical *Gata4* cofactor, which plays a crucial role in maintaining chamber-specific identity in both early- and post-differentiation of cardiomyocytes during cardiac morphogenesis in zebrafish [59,60]. In this study, we observed significant increases in *Nkx2.5* mRNA transcription level by 56%, 60%, and 72% under 2,6-DCNP, 2,6-DBNP, and 2,6-DINP exposures, respectively (Fig. 4). This is because zebrafish larvae increased *Nkx2.5* mRNA transcription expression to rescue the abnormal phenotype of cardiac caused by 2,6-DHNP exposures [61]. Similar results were reported by Huang et al. [62], as they found that acrylamide might recover heart development by increasing the transcription level of *Nkx2.5* mRNA.

Myosin light chain polypeptide 7 (*Myl7*) plays a crucial role in modulating cardiac development and contractility, therefore being a useful marker of cardiac muscle chamber distinction, development, and differentiation [63]. In this study, 2,6-DHNP exposures significantly reduced *Myl7* mRNA transcriptions by 30%, 85%, and 90% under 2,6-DCNP, 2,6-DBNP, and 2,6-DINP exposures, respectively (Fig. 4). This finding indicated that 2,6-DHNP exposures compromised cardiac contractility in zebrafish, leading to degeneration of myocardial tissue and atrophic thinning of the cardiac muscle. This supports the results of SV–BA distance, interpreting that 2,6-DHNPs can induce cardiac enlargement via decreasing cardiac contractility. A similar result was reported by Lu et al. [64] as they demonstrated that emamectin benzoate can induce cardiomegaly by decreasing the mRNA transcription level of *Myl7*.

T-box transcription factors (*Tbx*) play key roles in the development of embryonic mesoderm, and *Tbx5* is crucial for the correct differentiation of myocardium and chamber morphogenesis [65]. As a result, we found that 2,6-DHNP exposures did not significantly affect *Tbx5* mRNA transcription level (Fig. 4), indicating that 2,6-DHNP exposures did not activate responses in myocardium and chamber morphogenesis. A similar result was also found by Zhang et al. [66] as they proposed that dilated cardiomyopathy occurrence is associated with *Tbx5* loss-of-function mutation, which is consistent with the aforementioned results of SV–BA distance and histopathological experiments.

In short, these results suggest that 2,6-DHNP exposures impeded cardiac development by mediating the production of cardiac myocytes, recruiting more cells to the cardiogenic field, and resulting in compromised cardiac contractility during cardiomyogenesis, thus validating the 2,6-DHNP-induced cardiotoxicity at the early stage of heart development in zebrafish.

### 3.7 Impacts of 2,6-DHNP exposures on the cardiac function of zebrafish larvae

**Fig. 5:**
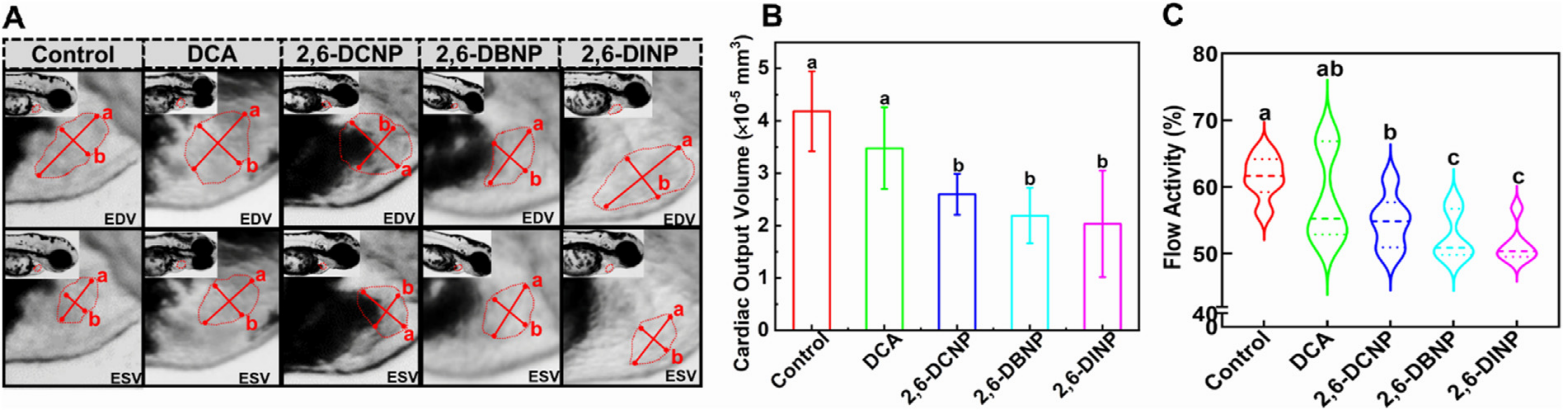
Impacts of 2,6-DHNPs and DCA exposures on the cardiac output (A and B) at 48 hpf and blood flow (C) at 72 hpf. (A) Heart dilatation and venous congestion images acquired at the diastolic stage of zebrafish heart beating under a dissecting stereomicroscope: “a” represents the long axis length of the myocardial borders of ventricles at diastole and systole, “b” represents short axis length of the myocardial borders of ventricles at diastole and systole, EDV represents end-diastolic volume, and ESV represents end-systolic volume. (B) The relative cardiac output of zebrafish larvae. Vertical bars represent ±SD, and different letters above bars indicate significant differences at *p* < 0.05.

The heart is the first definitive organ to develop and become functional in zebrafish larvae since any latter survival depends on its proper function [67]. Therefore, understanding the impacts of 2,6-DHNP exposures on cardiac function is important to assess their toxicological risks. In this study, we used heart rate, cardiac output, and blood flow as indicators to evaluate the impact of 2,6-DHNP exposures on cardiac function. While the result showed that 2,6-DHNP exposures did not affect the heart rate of zebrafish embryos, we observed a significant reduction in both cardiac output and blood flow under 2,6-DHNP exposures (Fig. 5). These results highlight the potential cardiac dysfunction caused by 2,6-DHNP exposures, even at SCs. This dysfunction, evident in reduced cardiac output and blood flow, suggests that the heart may not effectively support larval needs, thereby endangering larval survival [68]. Such observations align with another study [69], which revealed that prolonged and excessive cardiac overload can lead to decreased cardiac output and blood flow without altering the heart rate, potentially due to heart enlargement. Interestingly, the heart rate demonstrates distinct variations compared to the changes in cardiac output and blood flow. This disparity could be attributed to the protective role of zebrafish chorion, which shields the embryo from DHNP exposures. A similar case was reported as well [70], which highlighted the effectiveness of chorion as a barrier against bisphenol AF exposure in zebrafish larvae.

In short, our study revealed that 2,6-DHNP exposures can cause heart failure via decreasing cardiac output and blood flow, even at SCs. Given the integral role of the heart in numerous processes essential for tissue integrity and larval survival, its dysfunction can precipitate abnormal development or even death in zebrafish.

### 3.8 NAC mitigates 2,6-DHNP-induced cardiotoxicity in zebrafish embryos

Our results suggest that 2,6-DHNP-induced cardiac failure is caused by the ROS-apoptosis-cardiac anormogenesis pathway. This is evidenced by the increased ROS, apoptosis, and inhibited cardiac looping observed in zebrafish exposed to 2,6-DHNPs. To confirm the specific contribution of ROS, an effective ROS scavenger called NAC was co-exposed to zebrafish with 2,6-DHNPs. As a result, the ROS and apoptosis, which were expected to be induced by DHNPs, disappeared. Also, cardiac development-related gene and protein expressions, SV–BA distance, blood flow, and cardiac output returned to normal levels after the NAC addition (Table S4). Consistent with our results, Wang et al. also found that curcumin, another antioxidant, significantly inhibited ROS generation and reduced apoptosis, thus further alleviating the cardiotoxicity induced by a regulated DBP called chloroform in adult rats [71]. These results indicated that ROS is the key factor in 2,6-DHNP-induced cardiac failure, while the use of antioxidants may be beneficial in mitigating 2,6-DHNP-induced cardiotoxicity.

In short, ROS is a critical mediator of 2,6-DHNP-induced cardiac anormogenesis by triggering apoptosis. This underscores the role of ROS in the underlying mechanisms of 2,6-DHNP-induced cardiac failure in zebrafish and suggests the potential benefit of using antioxidants to counteract DHNP-induced cardiotoxicity. These insights are crucial for developing targeted interventions to mitigate the adverse effects of 2,6-DHNPs and similar compounds on cardiac development and function, ultimately contributing to improved public health and environmental safety.

## 4 Conclusion

Emerging aromatic DBPs—2,6-DHNPs have been identified in water samples from DWTPs and remain stubbornly resistant to HWTs. Despite their lower concentrations compared to regulated DBPs like trihalomethanes and haloacetic acids, their toxic effects are substantially more potent as they exert lethal toxicity 248 times greater than the regulated DBP. Furthermore, due to different halogen atoms, 2,6-DHNPs exhibited toxicities rank order of iodo-NP > bromo-NP > chloro-NP in generating ROS, induction apoptosis, and induced cardiotoxicity. Notably, exposure to 2,6-DHNPs at SCs, even as minimal as 19 μg/L, can trigger ROS production, promote apoptosis, as well as impair both cardiac looping and overall cardiac function in zebrafish. With 2,6-DHNP concentrations in various water sources reaching up to microgram per liter levels and being consumed by humans inevitably and regularly, there is a looming concern over its potentially detrimental impacts on public health. Nevertheless, the use of antioxidants might offer some relief by counteracting 2,6-DHNP-induced cardiotoxicity through the elimination of excess ROS.

## CRediT authorship contribution statement

Y.Y.L.: data curation, formal analysis, visualization, investigation, writing–original draft. C.X.W.: data curation, visualization, investigation, formal analysis. H.J.S., P.G.: conceptualization, supervision, writing–reviewing & editing, funding acquisition. L.Q. M., D.X.G.: formal analysis, writing–review & editing. H.C.H., H.Y.Y., H.J.L.: supervision and suggestions. X.F.H.: conceptualization, supervision, writing–reviewing & editing.

## Declaration of competing interests

The authors have declared no conflicts of interest.
