# Supplementary material for: Dihalogenated nitrophenols in drinking water: Prevalence, resistance to household treatment, and cardiotoxic impact on zebrafish embryo
Source: Eco Environ Health. 2024 Mar 4;3(2):183–91. doi: 10.1016/j.eehl.2024.02.004 (PMC11031730; doi:10.1016/j.eehl.2024.02.004)
Supplement: Multimedia component 1 [file mmc1.docx]

## Supplementary Material

## Dihalogenated nitrophenols in drinking water: Prevalence, resistance to household treatment, and cardiotoxic impact on zebrafish embryo

Hongjie Sun ^a,^*, Yingying Liu ^a^, Chunxiu Wu ^b^, Lena Q. Ma ^c^, Dongxing Guan ^c^, Huachang Hong ^a^, Haiying Yu ^a^, Hongjun Lin ^a^, Xianfeng Huang ^d^, Peng Gao ^e,f,^*

^a^ Key Laboratory of Watershed Earth Surface Processes and Ecological Security, College of Geography and Environmental Science, Zhejiang Normal University, Jinhua 321004, China

^b^ College of Chemistry and Life Science, Zhejiang Normal University, Jinhua 321004, China

^c^ Institute of Soil and Water Resources and Environmental Science, College of Environmental and Resource Sciences, Zhejiang University, Hangzhou 310058, China

^d^ National and Local Joint Engineering Research Center for Ecological Treatment Technology of Urban Water Pollution, College of Life and Environmental Science, Wenzhou University, Wenzhou 325035, China

^e^ Department of Environmental and Occupational Health, and Department of Civil and Environmental Engineering, University of Pittsburgh, Pittsburgh, PA 15261, United States

^f^ UPMC Hillman Cancer Center, Pittsburgh, PA 15232, United States

* Corresponding authors

E-mail addresses: [sunhj2016@zjnu.edu.cn](mailto:sunhj2016@zjnu.edu.cn) (H. Sun), [peg47@pitt.edu](mailto:peg47@pitt.edu) (P. Gao).

## Materials and Methods

### 1.1. Chemicals

2,6-dichloro-4-nitrophenol (2,6-DCNP, CAS: 618-80-4; purity 99%), 2,6-dibromo-4-nitrophenol (2,6-DBNP, CAS: 99-28-5; purity 99%), 2,6-diiodo-4-nitrophenol (2,6-DINP, CAS: 305-85-1; purity 99%) were obtained from Macklin Biochemical Co., Ltd (Shanghai, China). 2,6-DHNPs were dissolved in dimethyl sulfoxide (DMSO; Macklin, Shanghai), and blank DMSO was used as the control in each assay. 2, 7-dichlorofluorescein diacetate (DCFH-DA, CAS: 4091-99-0; purity 97%) was purchased from MedChemExpress Co., Ltd (Shanghai, China). Tricaine methanesulfonate (MS-222, CAS: 886-86-2; purity 98%) was purchased from Bide Pharmatech Ltd (Shanghai, China). Acridine Orange (AO) and paraformaldehyde (PFA, CAS: 50-00-0) were purchased from Sinopharm Chemical Reagent Co., Ltd (Shanghai, China). PBS was purchased from Shanghai Yuanfan Biotechnology Co. LTD (Shanghai, China). Tween-20 (CAS: 9005-64-5) and N,N-diethyl-p-phenylenediamine (DPD, CAS: 6283-63-2, purity 98%) were purchased from Shanghai Boer Chemical Reagent Co. LTD (Shanghai, China). Ascorbic acid (CAS: 50-81-7, purity 98%) was purchased from Sigma-Aldrich (St. Louis, MO, USA). N-Acetyl-L-cysteine (NAC) was from Shanghai Linen Technology Development Co., Ltd (Shanghai, China). Ethanol (CAS: 64-17-5; purity 99%) was purchased from Shanghai United Test Chemical Reagent Co., LTD (Shanghai, China). Dichloroacetic acid (DCA) and *o*-dianisidine (CAS: 119-90-4; purity 98%) were purchased from Aladdin Industrial Corporation (Shanghai, China). Optima LC/MS grade methyl tert-butyl ether (MtBE), methanol, and acetonitrile were from Sigma-Aldrich (Shanghai, China).

### 1.2. Zebrafish maintenance and embryo collection

The adult wild-type (AB strain) zebrafish and transgenic zebrafish (*cmlc*: EGFP) were bred in a recirculating system (ESEN, China). The zebrafish were maintained according to the method reported by our previous study [[1](#_ENREF_1)]. Briefly, the cultural parameters were set at 28 ± 0.5 °C, 14 h/10 h light/dark photoperiod, pH 7.2–7.5, dissolved oxygen concentration of 5–7 mg/L, and conductivity of 550 μS/cm. During acclimation, these zebrafish were fed with live brine shrimp every 12 hours. The male and female individuals were placed in a transparent incubator overnight, and spawning commenced the following morning. Obtained embryos were rinsed with embryonic rearing water, and the normal individuals were picked up using a stereoscopic microscope (SXZ7, Olympus, Japan). Healthy embryos were selected at the middle blastula stage (~4 hours post fertilization, hpf) for the following experiments.

### 1.3. Exposure experiments

2,6-DHNPs were dissolved in DMSO as the stock solution, and different concentrations of solutions were prepared by diluting the stock solution with the E3 medium. Before determining 2,6-DHNP toxicity, we explored the median lethal concentrations (LC_50_) of 2,6-DHNPs (2,6-DCNP, 2,6-DBNP, and 2,6-DINP) and DCA. The acute toxicity test of 2,6-DHNPs on the embryonic development of zebrafish was carried out according to our previous studies [[2](#_ENREF_2)]. Briefly, forty healthy embryos were placed in a weighted bottle, with each concentration having six replicates. The experiment was conducted at 28 ± 1 °C, 14 h light/10 h dark photoperiod, and pH 7.2–7.5. The experimental mediums were changed with 50% fresh solution daily to maintain constant solution concentrations. The experiment lasted for 120 hpf, and dead embryos were removed and recorded every 12 h. The LC_50_ values of 2,6-DHNPs and DCA were calculated using a three-parameter logistic model.

Considering that conventional toxicological studies usually apply high concentrations of pollutants [[3](#_ENREF_3),[4](#_ENREF_4)], the adverse effects induced by higher concentrations of pollutants surpass the detoxification abilities of organisms, resulting in co-occurring detrimental effects [[5](#_ENREF_5),[6](#_ENREF_6)]. As a result, current concerns about pollutant toxic potency are no longer solely based on lethal doses or LC_50_, but rather on SCs [[7](#_ENREF_7)]. The zebrafish embryos, therefore, were exposed to 2,6-DHNPs and DCA at SCs to assess their health risks. In the present study, each exposure medium and control group (E3 + 0.1% DMSO) were performed in six replicates, and the nominal exposure concentrations of 2,6-DHNPs were constantly verified and maintained (Supplementary Information). 2,6-DHNPs were dissolved in acetonitrile to prepare different concentrations of standard solutions, and the detected standard curve was listed in **Figure S1**. After exposure to 2,6-DHNPs at SCs for 120 h, zebrafish larvae were collected and rinsed with ice-cold physiological saline (0.68%, w/v) and stored at -80 °C for subsequent assays.

### 1.4. Health risks of 2,6-DHNPs and DCA in the zebrafish embryo

The toxicological risks assessment of 2,6-DHNPs in the early life stage of zebrafish was conducted [[8](#_ENREF_8)]. In the experiments, the zebrafish embryos were observed at 24, 48, 72, 96, and 120 hours post fertilization (hpf). Embryos without visible heartbeats and spontaneous movements, lack of somite formation or coagulation, and larvae with coagulation or cardiac arrest were defined as the endpoint of mortality. The malformation rate of each treatment was calculated every 24 hours. Pericardial edema, yolk sac edema, and tail malformation were defined as the endpoint of malformation. In addition, the hatching rate, which is defined as the complete separation of the larvae from the egg membrane, was calculated at 72 hpf.

Spontaneous tail coiling analysis was conducted [[9](#_ENREF_9)]. Briefly, normal embryos (at 24 hpf) in each treatment were randomly collected to take 1 min videos at 0.8 × magnification under the microscope (SXZ7, Olympus, Japan). Analysis was performed using the DanioScope Software (Noldus, Netherlands), and spontaneous tail coiling activity was expressed as the proportion of the time of flicked tail in 1 min (%). The proportion of the time of spontaneous head-tail contraction of each embryo was counted, with the average for each group being calculated. Experiments were performed in duplicate, with 15 embryos per treatment.

Heart rate and cardiac output analysis were conducted [[1](#_ENREF_1),[10](#_ENREF_10)]. Briefly, 10 embryos (at 48 hpf) in each treatment were placed on a slide to assess the heart rate and cardiac output under the microscope for videos (SXZ7, Olympus, Japan) after anesthetizing (0.168 mg/mL MS-222) for 1 min. Heart rate was measured using the DanioScope software (Noldus, Netherlands) according to the changes in pixels on a frame-by-frame, which is reported as the number of beats per second at 48 hpf by DanioScope. Subsequently, images from videos were used to measure the long axis length (a) and short axis length (b) between the myocardial borders of ventricles at diastole and systole, respectively. End-diastolic volume (EDV) and end-systolic volume (ESV) of ventricles were calculated according to the formula: volume = 4/3 πab^2^. The stroke volume of the ventricular was then calculated using the formula: stroke volume = EDV – ESV, and cardiac output was estimated by multiplying stroke volume by heart rate [10].

Blood flow was also evaluated [[1](#_ENREF_1)]. Briefly, 10 zebrafish larvae (72 hpf) in each treatment were placed on a slide to assess the blood flow under a microscope for videos (SXZ7, Olympus, Japan), after anesthetizing (0.168 mg/mL MS-222) for 1 min. Blood flow was measured according to the changes in pixels on a frame-by-frame, which is the percentage of activity within that area (%) at 72 hpf by DanioScope.

Body length was measured from the anterior portion of the larva to the tip of the tail, which was assayed by the DanioScope Software [[8](#_ENREF_8)]. Briefly, 8 larvae (at 120 hpf) in each treatment were transferred to a microscope slide with scale and photographed (SXZ7, Olympus, Japan) after anesthetizing (0.168 mg/mL MS-222) for 1 min.

### 1.5. Analysis of malondialdehyde and 8-hydroxydeoxyguanosine

Malonaldehyde (MDA), a final product of tissue lipid peroxidation, can thus be used as an indicator of oxidative damage of lipids by ROS, which was analyzed using a diagnostic reagent kit (Jiancheng, Nanjing, China) according to the instructions. Briefly, approximately sixty larvae (at 120 hpf) for each treatment group were washed with cold physiological saline (0.68%, w/v) and homogenized in cold physiological saline solution (larvae/total was 4%). This homogenate was centrifuged at 4000 rpm at 4 °C for 10 min, and the supernatants were transferred to clean tubes for MDA and protein analysis. All samples were kept on ice throughout the experiment.

8-hydroxydeoxyguanosine (8-OHdG) is a well-established marker for assessing DNA damage caused by oxidative stress. It is formed as a result of ROS-induced damage to 2'-deoxyguanosine, a component of the DNA backbone. In this study, the levels of 8-OHdG were measured using an enzyme-linked immunosorbent assay, a sensitive and specific method for quantifying this marker. Approximately 100 larvae were ground in phosphate buffer solution (larvae/total was 10%). After centrifuging for 20 min at 3000 rpm, supernatants were collected for the following color reaction. The optical density of each sample was analyzed at 450 nm within 10 minutes. 8-OHdG level was determined based on the standard curve and expressed in arbitrary units (ng/mg protein).

### 1.6. Determination of cardiac tissue damage caused by 2,6-DHNPs and DCA using hematoxylin-eosin staining

Hematoxylin and eosin staining were applied for histopathological evaluations. After exposure for 120 hpf, three zebrafish larvae of each group were firstly rinsed with E3 medium, then fixed with 4% paraformaldehyde solution overnight, and processed with a series of procedures, including embedding in paraffin, sectioning, hematoxylin, and eosin staining. Last, histopathological changes were recorded using a microscope (BX43, Olympus, Japan).

### 1.7. Determination of mRNA transcriptions related to cardiac development using qPCR

Isolation of RNA and synthesis of complementary DNA were carried out[[](#_ENREF_1)1]. Briefly, total RNA was isolated from the larvae using a homogenizer with TRIzol reagent according to the manufacturer's protocols (TaKaRa, Japan). After quantitating with a NanoDrop spectrophotometer (One, Thermo Scientific, USA), 1 μg total RNA was reverse transcribed using reverse transcriptase and random primers (TaKaRa, Japan). Expression of the target genes and internal control were measured by quantitative real-time polymerase chain reaction (qRT-PCR). All PCR (25 μL) reactions comprised 12.5 μL of SYBR Premix Ex TaqII (TaKaRa), 0.5 μL cDNA, 10 pmol of each forward and reversed primer (**Table S1**), and 11 μL ultrapure water. The thermal cycling profile was set at 95 °C for 30 s, 40 cycles of 95 °C for 5 s, and 60 °C for 30 s. Fluorescence yields obtained from three replicate reactions of each cDNA sample were analyzed using the real-time fluorescent quantitative PCR instrument (LightCycler96, Roche, Switzerland). The *β-actin* was used as an internal reference, and the fold changes of target genes were computed using the 2^-ΔΔCt^ method[[11](#_ENREF_11)].

### 1.8. Determination of cardiac-related protein expressions using western blotting

Caspase-3 is an important factor in the establishment of apoptosis and is an important apoptosis-activated enzyme of the caspase family [[12](#_ENREF_12)], while myocyte enhancer factor 2 (Mef2c) has been linked with early heart development [[13](#_ENREF_13)], and glyceraldehyde 3-phosphate dehydrogenase (GAPDH) was used as a reference control protein in this study. Their expression levels were analyzed by western blot [[14](#_ENREF_14)]. Approximately 20 mg larvae were homogenized with 100 μL lysis buffer and centrifuged at 10,000 rpm for 15 minutes at 4 °C, and then the supernatants were collected as protein extract. Supernatants were electrophoresed on 10% sodium dodecyl sulfate–polyacrylamide gel electrophoresis and transferred to polyvinylidene fluoride membranes, which were then blocked with 5% non-fat milk for 1 h at room temperature. The membrane was incubated with the antibodies of Caspase-3 (1: 1000, ab13847, Abcam, England) and Mef2c (1: 1000, BS-4130R, Bioss, China) overnight at 4°C, which were then washed by PBST (1× PBS and 0.1% Tween-20) and incubated with a horseradish peroxidase-conjugated anti-rabbit Ig G secondary antibody (1:5000, D110058-0100, Sangon, China) for 1 h at room temperature. After washing three times with PBST, the mean integrated positive optical densities of Caspase-3 and Mef2c were obtained using a scanner (Tanon-6600, Shanghai, China). Densitometry measurements of band intensity in the western blots were performed using Image J (Bethesda, USA). For the western blotting assay, each sample comprised three replicates.

### 1.9. Determination of 2,6-DHNPs in exposure solutions

2,6-DHNP solutions were prepared for liquid chromatography coupled with mass spectrometry analysis. First, the pH of 500 mL of each solution was adjusted to 0.5 using 7:3 (v/v) concentrated sulfuric acid/water and 100 g Na_2_SO_4_. After extraction with 50 mL of methyl *tert*-butyl ether (MtBE), the MtBE layer of each sample was settled into 0.5 mL through rotary and nitrogen evaporators. Subsequently, the 0.5 mL solution in MtBE was mixed with 10 mL acetonitrile and then evaporated to obtain 1 mL. Before injection, 1 mL of the solution was diluted 100-fold with methanol and filtered through a 0.22 μm PTFE filter (PALL, US).

The 2,6-DCNP and 2,6-DBNP exposure solutions were analyzed in full scan mode using a SCIEX TripleTOF 4600 mass spectrometry operating in the negative electrospray ionization (ESI) mode. Before that, a pretreated sample (5 μL) was injected into ultra-performance liquid chromatography (UPLC). The UPLC separation was performed with an HSS T3 column (100 × 2.1 mm, 1.8 μm particle size, Waters). The gradient eluent was constituted with methanol/water, which changed from 5/95 (v/v) to 95/5 linearly in the first 8 min, returned to 5/95 in 0.1 min, and further maintained for 11 minutes for the re-equilibrating column. The flow rate was 0.40 mL/min, and the column temperature was 35 °C. A full scan time-of-flight (TOF) survey (accumulation time 0.5 ms; m/z: 100–500) was acquired using the following parameters: curtain gas, 35 psi; nebulizer and heated gases, 50 psi; ion spray voltage, -4500 V; ion source temperature, 500 °C; declustering potential, -100 V; and collision energy, -10 V. Standard solutions of 1, 2, 5, 10, 50, and 80 μg/L 2,6-DCNP and 2,6-DBNP were determined according to the chromatographic conditions. The peak area was regarded as the vertical coordinate and the concentration was taken as the horizontal coordinate to draft the standard curve for quantification (**Figure S1A and 1B**). Standard solutions of 1, 3, 5, 7, and 8 μg/L 2,6-DCNP and 1, 2, 4, 6, and 8 μg/L 2,6-DBNP were determined according to the chromatographic conditions for DWTPs testing (**Figure S4A**). Standard solutions of 1, 2, 2.5, 3, 4, and 8 μg/L 2,6-DCNP and 1, 2, 4, 6, and 7 μg/L 2,6-DBNP were determined according to the chromatographic conditions for tap water testing (**Figure S4B**). Standard solutions of 1, 2, 5, 10, 20, 50, and 80 μg/L 2,6-DCNP and 2, 5, 10, 20, 50, and 80 μg/L 2,6-DBNP were determined according to the chromatographic conditions for tap water which spiked 50 μg/L 2,6-DHNPs standards testing (**Figure S4C**). All the 2,6-DCNP and 2,6-DBNP samples were duplicated.

The 2,6-DINP exposure solution was analyzed using an Agilent 6495 LC-QqQ. The HPLC system was coupled to an Agilent triple quadrupole mass spectrometer (MS) with an Agilent jet-stream electrospray ion source (AJS-ESI). The UPLC separation was performed with an HSS T3 column (100 × 2.1 mm, 1.8 μm particle size, Waters). A pretreated sample (5 μL) was injected into UPLC. The gradient eluent consisted of methanol/water, which changed from 10/90 (v/v) to 90/10 linearly in the first 5 min, returned to 10/90 linearly in 19 min, and was further maintained for 25 minutes for re-equilibration of the column. The flow rate was 0.30 mL/min, and the column temperature was 40 °C. Tandem mass spectrometry (MS/MS) data were acquired under the negative ion mode. The MS parameters were optimized and set as follows: fragmentation voltage, 166 V; collision energy, 60 V; drying gas (nitrogen) temperature, 180 °C; drying gas flow rate 13 L/min; nebulizer pressure, 40 psi; sheath gas temperature, 400 °C; sheath gas flow, 12 L/min; and capillary voltage, 3500 V. Based on this analysis method in the Multiple Reaction Monitoring mode, 389.9-262.9/127 was selected as the monitoring object. Based on the chromatographic conditions, 1, 2, 5, 10, 30, and 50 μg/L 2,6-DINP standard solutions were prepared. The peak area was regarded as the vertical coordinate and the concentration was regarded as the horizontal coordinate to construct the standard curve for quantification (**Figure S1C**). Standard solutions of 1, 2, 5, 10, 20, 50, and 80 μg/L 2,6-DINP were determined according to the chromatographic conditions for tap water which spiked 50 μg/L 2,6-DHNP standards testing (**Figure S4C**). All the 2,6-DINP samples were duplicated.

**References**

[1] Dong M, Wang J, Liu Y, He Q, Sun H, Xu Z, Hong H, Lin H, Gao P. 3-Bromocarbazole-Induced Developmental Neurotoxicity and Effect Mechanisms in Zebrafish. ACS ES&T Water. 2023, DOI: 10.1021/acsestwater.3c00108.

[2] Sun H-J, Zhang Y, Zhang J-Y, Lin H, Chen J, Hong H. The toxicity of 2,6-dichlorobenzoquinone on the early life stage of zebrafish: A survey on the endpoints at developmental toxicity, oxidative stress, genotoxicity and cytotoxicity. Environmental Pollution. 2019, 245: 719-724.

[3] Deng Y, Zhang Y, Zhang R, Wu B, Ding L, Xu K, Ren H. Mice in vivo toxicity studies for monohaloacetamides emerging disinfection byproducts based on metabolomic methods. Environmental science & technology. 2014, 48(14): 8212-8218.

[4] Shen R, Yu Y, Lan R, Yu R, Yuan Z, Xia Z. The cardiovascular toxicity induced by high doses of gatifloxacin and ciprofloxacin in zebrafish. Environmental Pollution. 2019, 254: 112861.

[5] Wen H, Dan M, Yang Y, Lyu J, Shao A, Cheng X, Chen L, Xu L. Acute toxicity and genotoxicity of silver nanoparticle in rats. PloS one. 2017, 12(9): e0185554.

[6] Li H, Yu S, Cao F, Wang C, Zheng M, Li X, Qiu L. Developmental toxicity and potential mechanisms of pyraoxystrobin to zebrafish (Danio rerio). Ecotoxicology and environmental safety. 2018, 151: 1-9.

[7] Chapman PM, Fairbrother A, Brown D. A critical evaluation of safety (uncertainty) factors for ecological risk assessment. Environmental Toxicology and Chemistry. 1998, 17(1): 99-108.

[8] Wang W-Q, Chen H-H, Zhao W-J, Fang K-M, Sun H-J, Zhu F-Y. Ecotoxicological assessment of spent battery extract using zebrafish embryotoxicity test: A multi-biomarker approach. Chemosphere. 2022, 287: 132120.

[9] Zhang S, Ye C, Zhao W, An L, Yu X, Zhang L, Sun H, Feng M. Product identification and toxicity change during oxidation of methotrexate by ferrate and permanganate in water. Frontiers of Environmental Science & Engineering. 2021, 16(7): 93.

[10] Chen Z, Huang W, Dahme T, Rottbauer W, Ackerman MJ, Xu X. Depletion of zebrafish essential and regulatory myosin light chains reduces cardiac function through distinct mechanisms. Cardiovascular Research. 2008, 79(1): 97-108.

[11] Livak KJ, Schmittgen TD. Analysis of relative gene expression data using real-time quantitative PCR and the 2− ΔΔCT method. methods. 2001, 25(4): 402-408.

[12] Zhao X, Ren X, Zhu R, Luo Z, Ren B. Zinc oxide nanoparticles induce oxidative DNA damage and ROS-triggered mitochondria-mediated apoptosis in zebrafish embryos. Aquatic Toxicology. 2016, 180: 56-70.

[13] Hinits Y, Pan L, Walker C, Dowd J, Moens CB, Hughes SM. Zebrafish Mef2ca and Mef2cb are essential for both first and second heart field cardiomyocyte differentiation. Developmental Biology. 2012, 369(2): 199-210.

[14] Sun H-J, Ding S, Guan D-X, Ma LQ. Nrf2/Keap1 pathway in countering arsenic-induced oxidative stress in mice after chronic exposure at environmentally-relevant concentrations. Chemosphere. 2022: 135256.

**Table S1.** Primer sequence for the quantitative reverse transcription-polymerase chain reaction used in this study.

| Gene | Forward primer (5' to 3') | Reverse primer (5' to 3') |
| --- | --- | --- |
| *β-Actin* | ATGGATGAGGAAATCGCTGCC | CTCCCTGATGTCTGGGTCGTC |
| *Nkx2.5* | ATGCCATCCGGATCCTCTCT | GTCTTCCTGACAACAGCCGA |
| *Gata5* | AGACAAGCCTCGGTGACAAG | AATATCGCCGTCGAGGTTCC |
| *Myl7* | AGGCTCTTCCAATGTCT | GGTTTCCTCTGGGTCTG |
| *Tbx5* | ATTCGCCGATAACAAATGG | CGCCTTGACGATGTGGAT |
| *Gata4* | GCCGGGATACCGCAACTTAT | AGTACGGAGCTGTCGAAGTG |

**Table S2.** The 2,6-DHNP concentrations in water samples from each treatment process of two DWTPs.

| Sample Site | Treatment | 2,6-DCNP (ng/L) | 2,6-DBNP (ng/L) | 2,6-DINP (ng/L) |
| --- | --- | --- | --- | --- |
| DWTP A | Influent Water | 1.37±0.06 | 1.59±0.05 | ND |
|  | Chlorination | ― | ― | ― |
|  | Primary Precipitation | 1.87±0.05 | 2.11±0.09 | ND |
|  | Secondary Precipitation | 2.36±0.07 | 1.89±0.06 | ND |
|  | Primary Filtration | 2.43±0.02 | 1.62±0.03 | ND |
|  | Secondary Filtration | 2.28±0.04 | 1.94±0.06 | ND |
|  | Effluent Water | 2.32±0.07 | 2.16±0.01 | ND |
| DWTP B | Influent Water | 1.80±0.04 | 1.95±0.06 | ND |
|  | Chlorination | ― | ― | ― |
|  | Primary Precipitation | 2.13±0.04 | 1.60±0.07 | ND |
|  | Secondary Precipitation | 2.38±0.05 | 1.44±0.00 | ND |
|  | Primary Filtration | 2.76±0.05 | 1.85±0.02 | ND |
|  | Secondary Filtration | 2.65±0.02 | 2.98±0.05 | ND |
|  | Effluent Water | 2.74±0.03 | 2.71±0.03 | ND |

ND, Not detected.

**Table S3.** Water quality parameters of different treatment samples in DWTP A and DWTP B. A1 to A6 represent the different treatment samples in DWTP A, and B2 to B6 represent the different treatment samples in DWTP B. C represents the tap water from ZJNU.

| Treatment Technology | Temp  (°C) | TDS  (mg/L) | Cand  (μs/cm) | DOC  (mg/L) | UVA_254_  (/cm) | SUVA_254_  (L/mg/m) | pH | Cl^-^  (mg/L) | Br^-^  (μg/L) | I^-^  (μg/L) | TN  (mg/L) | NO_3_-N  (mg/L) | NH_3_-N  (mg/L) | NO_2_-N  (mg/L) | Residual chlorine  (mg/L) |
| --- | --- | --- | --- | --- | --- | --- | --- | --- | --- | --- | --- | --- | --- | --- | --- |
| A1 | 18.2 | 22.6 | 45.3 | 0.56 | 0.014 | 2.53 | 6.8 | 3.32 | 16.17 | ND | 1.16 | 0.88 | 0.03 | ND | ND |
| A2 | 16.7 | 25.7 | 51.4 | 0.45 | 0.012 | 2.54 | 7.0 | 3.42 | 16.84 | ND | 1.17 | 0.86 | 0.04 | ND | 0.06 |
| A3 | 16.9 | 25.6 | 51.5 | 0.44 | 0.006 | 1.24 | 7.0 | 3.40 | 16.03 | ND | 1.19 | 0.82 | 0.06 | ND | 0.05 |
| A4 | 15.9 | 25.3 | 50.6 | 0.93 | 0.006 | 0.59 | 6.9 | 3.64 | 16.57 | ND | 1.18 | 0.84 | 0.05 | ND | 0.32 |
| A5 | 17.9 | 25.3 | 50.6 | 0.42 | 0.004 | 0.96 | 7.0 | 3.69 | 16.64 | ND | 1.17 | 0.85 | 0.04 | ND | 0.35 |
| A6 | 18.2 | 26.2 | 52.3 | 0.58 | 0.007 | 1.13 | 7.3 | 3.82 | 16.87 | ND | 1.18 | 0.89 | 0.05 | ND | 0.54 |
| B1 | 16.1 | 23.3 | 46.6 | 0.51 | 0.012 | 2.35 | 6.6 | 3.35 | 14.30 | ND | 1.12 | 0.86 | 0.03 | ND | ND |
| B2 | 14.3 | 26.6 | 52.6 | 0.47 | 0.005 | 0.96 | 7.0 | 3.39 | 14.64 | ND | 1.17 | 0.86 | 0.04 | ND | ND |
| B3 | 14.6 | 27.0 | 53.3 | 0.42 | 0.004 | 0.84 | 7.3 | 3.39 | 14.42 | ND | 1.16 | 0.85 | 0.03 | ND | ND |
| B4 | 14.6 | 26.8 | 53.9 | 0.44 | 0.008 | 1.71 | 7.3 | 3.62 | 14.81 | ND | 1.17 | 0.84 | 0.04 | ND | 0.28 |
| B5 | 15.1 | 26.8 | 53.7 | 0.42 | 0.007 | 1.53 | 7.2 | 3.68 | 14.13 | ND | 1.16 | 0.87 | 0.03 | ND | 0.26 |
| B6 | 15.0 | 27.1 | 54.1 | 0.46 | 0.003 | 0.54 | 7.2 | 3.75 | 14.94 | ND | 1.16 | 0.87 | 0.03 | ND | 0.43 |
| C | 18.1 | 26.4 | 53.5 | 0.49 | 0.005 | 1.02 | 7.3 | 3.78 | 15.13 | ND | 1.16 | 0.87 | 0.03 | ND | 0.39 |

A1: Influent water, A2: Primary precipitation water, A3: Secondary precipitation water,

A4: Primary filtration water, A5: Secondary filtration water, A6: Effluent water.

B1: Influent water, B2: Primary precipitation water, B3: Secondary precipitation water,

B4: Primary filtration water, B5: Secondary filtration water, B6: Effluent water.

C: Control.

**Table S4.** The changes in ROS levels, apoptosis levels, SV-BA distances, blood flow, cardiac output, and Mef2c protein and five genes (*Gata4, Myl7, Tbx5,* *Gata5*, and *Nkx2.5*) mRNA transcriptional expressions in zebrafish larvae after co-exposures to DCA, 2,6-DHNPs, and NAC. Different letters indicate significant differences at *p* < 0.05.

|  | ROS  (Fluorescence intensity) | AO Staining  (Fluorescence intensity ) | SV-BA distance (μm) | Blood Flow  (%) | Cardiac  Output  (* 10^-5^ mm^3^) | MEF2C  (Relative protein expression) | Gata4  (Relative mRNA level ) | Myl7  (Relative mRNA level) | Tbx5  (Relative mRNA level) | Gata5  (Relative mRNA level) | Nkx2.5  (Relative mRNA level) |
| --- | --- | --- | --- | --- | --- | --- | --- | --- | --- | --- | --- |
| Control | 1.53^a^ (±0.87) | 0.50 ^a^ (±0.17) | 146.99 ^a^ (±4.04) | 61.52 ^a^ (±3.24) | 4.18 ^a^ (±1.76) | 2.031^a^ (±0.46) | 1 ^a^ | 1 ^a^ | 1 ^a^ | 1 ^a^ | 1 ^a^ |
| DCA  +NAC | 1.62 ^a^ (±0.72) | 0.51 ^a^ (±0.17) | 147.12 ^a^ (±1.51) | 58.33 ^a^ (±7.29) | 3.58 ^a^ (±1.04) | 1.73 ^a^ (±0.54) | 0.68 ^a^  (±0.36) | 0.73 ^a^  (±0.45) | 1.20 ^a^  (±1.33) | 0.98 ^a^  (±1.44) | 0.16 ^a^  (±0.12) |
| 2,6-DCNP  +NAC | 1.81 ^a^ (±0.64) | 0.54 ^a^ (±0.28 | 147.69 ^a^ (±4.09) | 55.04 ^a^ (±3.96) | 3.89 ^a^ (±0.75) | 2.05 ^a^ (±0.87) | 0.81 ^a^ (±0.59) | 0.72 ^a^  (±0.46) | 1.24 ^a^ (±0.42) | 0.90 ^a^ (±0.29) | 0.32 ^a^  (±0.17) |
| 2,6-DBNP  +NAC | 1.76 ^a^ (±0.89) | 0.54 ^a^ (±0.25) | 150.84 ^a^ (±1.80) | 56.89 ^a^ (±8.15) | 4.03 ^a^ (±0.90) | 2.10 ^a^ (±0.93) | 1.61 ^a^  (±2.16) | 0.64 ^a^  (±0.22) | 1.250^a^  (±0.53) | 0.66 ^a^  (±0.29) | 0.76 ^a^  (±0.47) |
| 2,6-DINP  +NAC | 1.65 ^a^ (±0.96) | 0.48 ^a^ (±0.21) | 151.14 ^a^ (±2.49) | 54.92 ^a^ (±4.70) | 3.54 ^a^ (±0.63) | 2.32 ^a^ (±0.74) | 1.58 ^a^  (±2.17) | 0.78 ^a^  (±0.25) | 1.54 ^a^  (±1.62) | 0.24 ^a^  (±0.06) | 0.98 ^a^  (±0.73) |


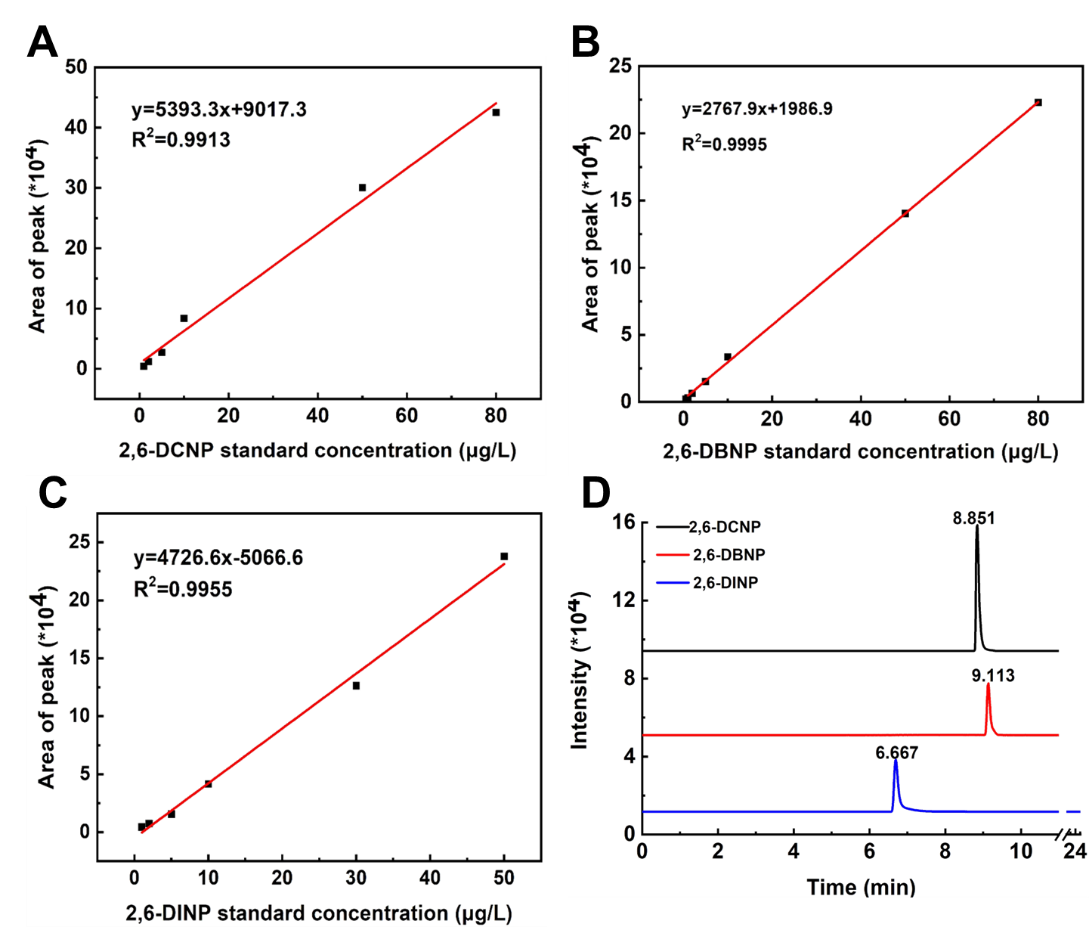


**Figure S1.** Standard quantification curves of 2,6-DCNP (A), 2,6-DBNP (B), 2,6-DINP (C), and liquid chromatogram of the determined 2,6-DHNPs (D). Duplicate experiments were performed for each concentration.


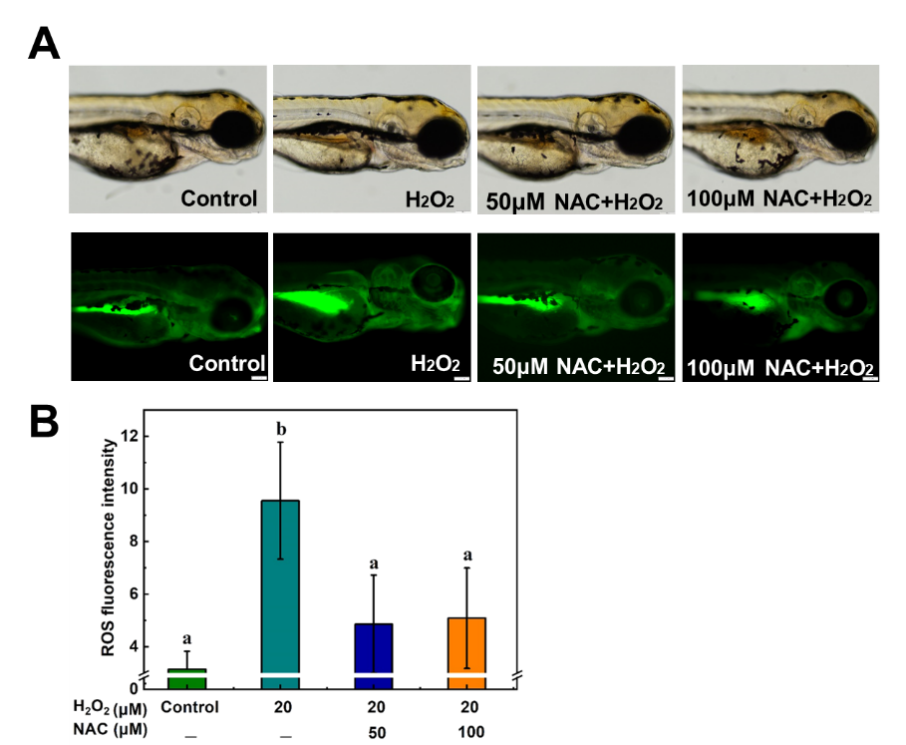


**Figure S2.** *In vivo* assessment of NAC optimal concentrations in zebrafish larvae at 72 hpf. (A) The distribution of fluorescence visualizing ROS in zebrafish larvae exposed to control, H_2_O_2_, and with the additions of 50 μM NAC and 100 μM NAC, respectively. (B) The fluorescence intensity of ROS after exposure to control, H_2_O_2_, and with the additions of 50 μM NAC and 100 μM NAC, respectively (n=3).


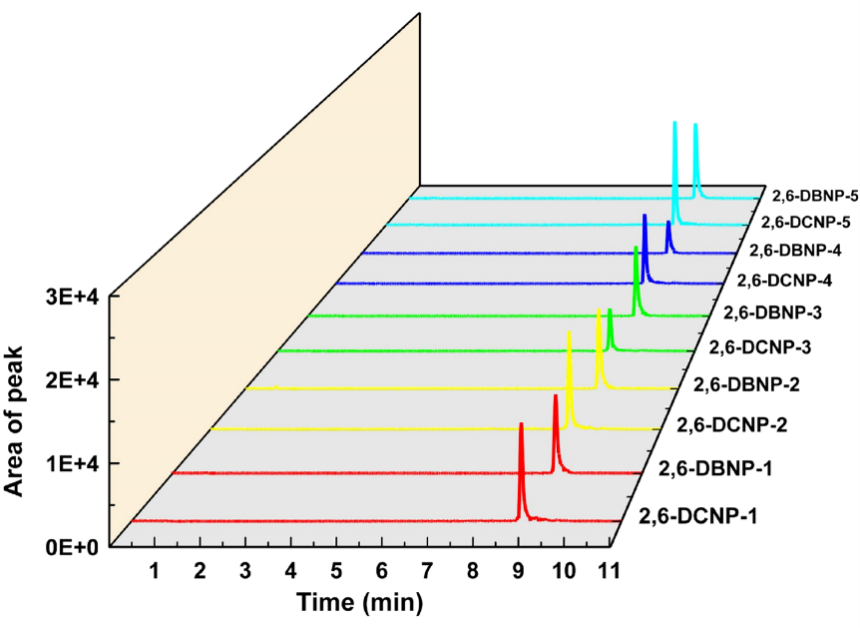


**Figure S3.** Representative chromatogram of 2,6-DCNP, 2,6-DBNP, and 2,6-DINP in the water samples after household water treatments. 1: Tap water; 2: Boiled water; 3: Filtered water, 4: Microwave heated water; 5: Ultrasound-treated water (n = 2).


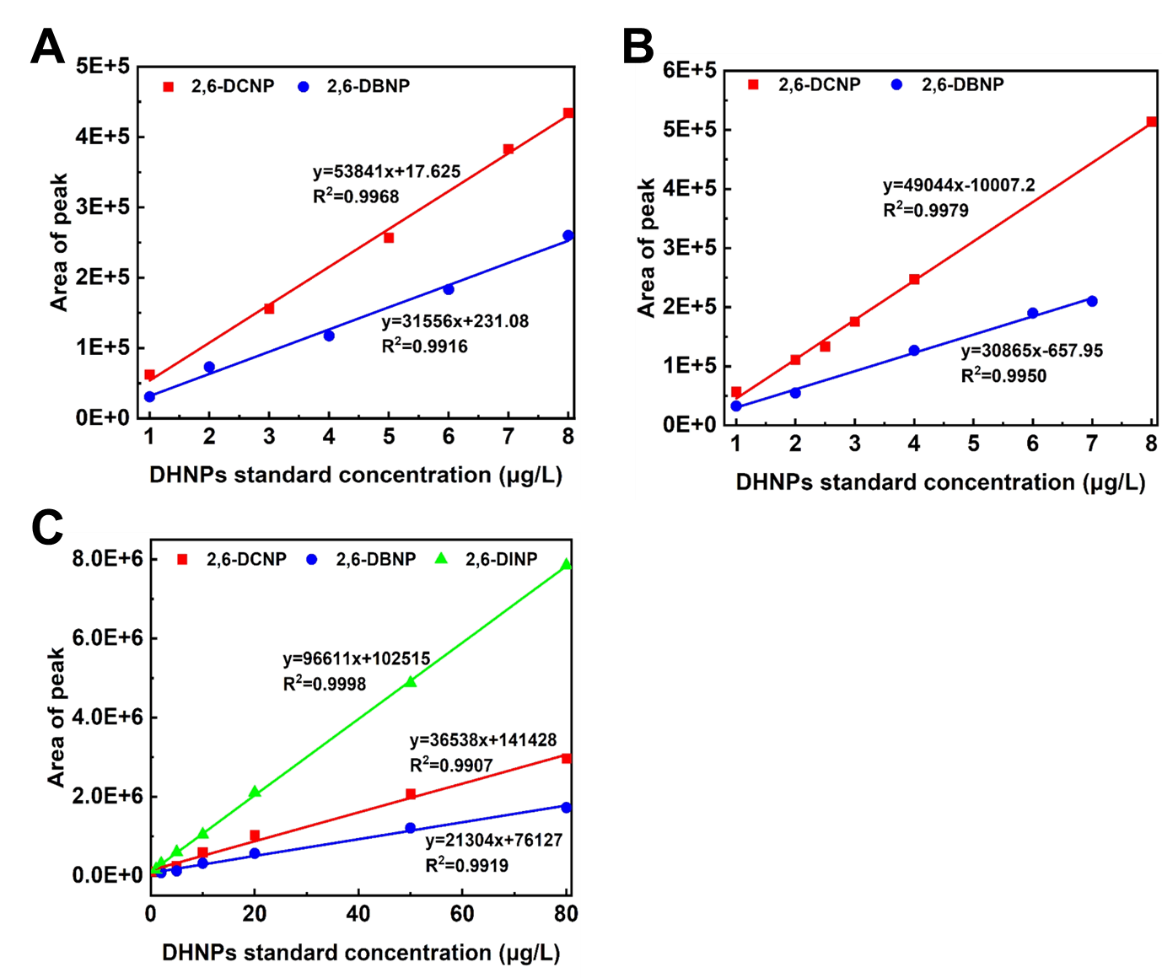


**Figure S4.** Standard quantification curves of 2,6-DHNPs (μg/L) in DWTPs water (A), tap water (B), and 50 μg/L spiked in tap water (C). Duplicate experiments were performed for each concentration.
